# A Pragmatic Approach to Pancreatic Trauma: A Single-Center Experience From a Tertiary Care Center

**DOI:** 10.7759/cureus.24793

**Published:** 2022-05-06

**Authors:** RDR Somasekar, Pothugunta S Krishna, B Kesavan, A Siva Sankar

**Affiliations:** 1 Surgical Gastroenterology, Government Mohan Kumaramangalam Medical College Hospital, Salem, IND

**Keywords:** whipple's procedure, central pancreatectomy, step up approach, kimura's procedure, pancreatic trauma

## Abstract

Introduction

Pancreatic trauma is rare and is usually associated with adjacent organ and vascular injuries, which adds to the high morbidity and mortality. In the American Association for the Surgery of Trauma (AAST) pancreatic trauma (PT) grading system, the higher grades are a composite of less and more severe extents of injuries. We hereby present an observational study of PT with management based on an indigenous algorithmic approach. Our protocol incorporating both the extent of disruption of the main pancreatic duct (MPD) and its amenability to interventions (endoscopic, radiological, or surgical) is pragmatic.

Methods

Ours is a retrospective observational study of 28 consecutive cases of PT, done over a three-year period in an academic institution, by an expert Surgical Gastroenterology unit. All patients diagnosed with PT on a contrast abdominal CT scan were included. After stabilization, they were stratified and managed according to an indigenous protocol. The primary outcome measure was treatment success in terms of recovery. The secondary outcome measure was morbidity of any form.

Results

One patient with Grade 1 PT was operated on for associated hollow viscus injury. Two patients with AAST Grade 2 and two patients with AAST Grade 3 injury were managed successfully without surgery. Twelve of 21 patients with Grade 3 PT underwent Kimura’s splenic vessel preserving distal pancreatectomy. Distal pancreatectomy with splenectomy and central pancreatectomy with Roux-en-Y pancreaticojejunostomy (PJ) was done for 7/21 and 2/21 patients, respectively, with Grade 3 PT. Two with Grade 5 injury underwent trauma Whipple. The overall mortality and morbidity rates in our series were 15.7% and 64%, respectively.

Conclusion

The pathogenesis in PT is a dynamic process and shows temporal evolution. These patients require serial and periodical clinical and radiological monitoring, especially in those managed conservatively initially. PT can be low or high grade. Patients with isolated low-grade PT can be managed according to the standard step-up approach for acute pancreatitis. A carefully selected subgroup of patients with partial MPD disruption either in the head or body of the pancreas can be managed by endotherapy. Complete distal parenchymal transections require early surgery tailored to individual patients in the form of either splenic vessel preserving distal pancreatectomy (SPDP) or distal pancreatectomy with splenectomy (DP+S). Damage control surgery is the dictum in unstable patients with Grades 4 and 5 injuries not responding to resuscitative measures. A trauma Whipple can be done in a carefully selected subgroup of stable patients with proximal massive disruptions in an experienced hepato-pancreatico-biliary (HPB) unit.

## Introduction

Pancreatic trauma is rare, with an incidence of 0.2% of all blunt abdominal injuries and 1.1% of penetrating abdominal injuries [1]. It is associated with a high incidence of injury to adjacent organs and major vascular structures, which adds to the high morbidity and mortality [2]. Mortality and morbidity may range from 12-33% and 25-50% respectively [3]. Isolated pancreatic injury is rare and in the majority of cases (90%) involves at least one other abdominal organ [4]. Early mortality is due to exsanguination owing to associated vascular injuries, while late mortality is attributable to sepsis and multiorgan dysfunction [5]. Timely diagnosis and management are of prime importance to reduce morbidity and mortality [6].

The most widely accepted classification and grading system for pancreatic trauma is the American Association for the Surgery of Trauma (AAST) system, which is based on the site of damage to the pancreatic parenchyma and the ductal system [7]. There are several options for the management of PT, ranging from expectant management to operative procedures [8]. We hereby present an observational study of PT with management based on an indigenous algorithmic approach.

## Materials and methods

**Figure 1:**
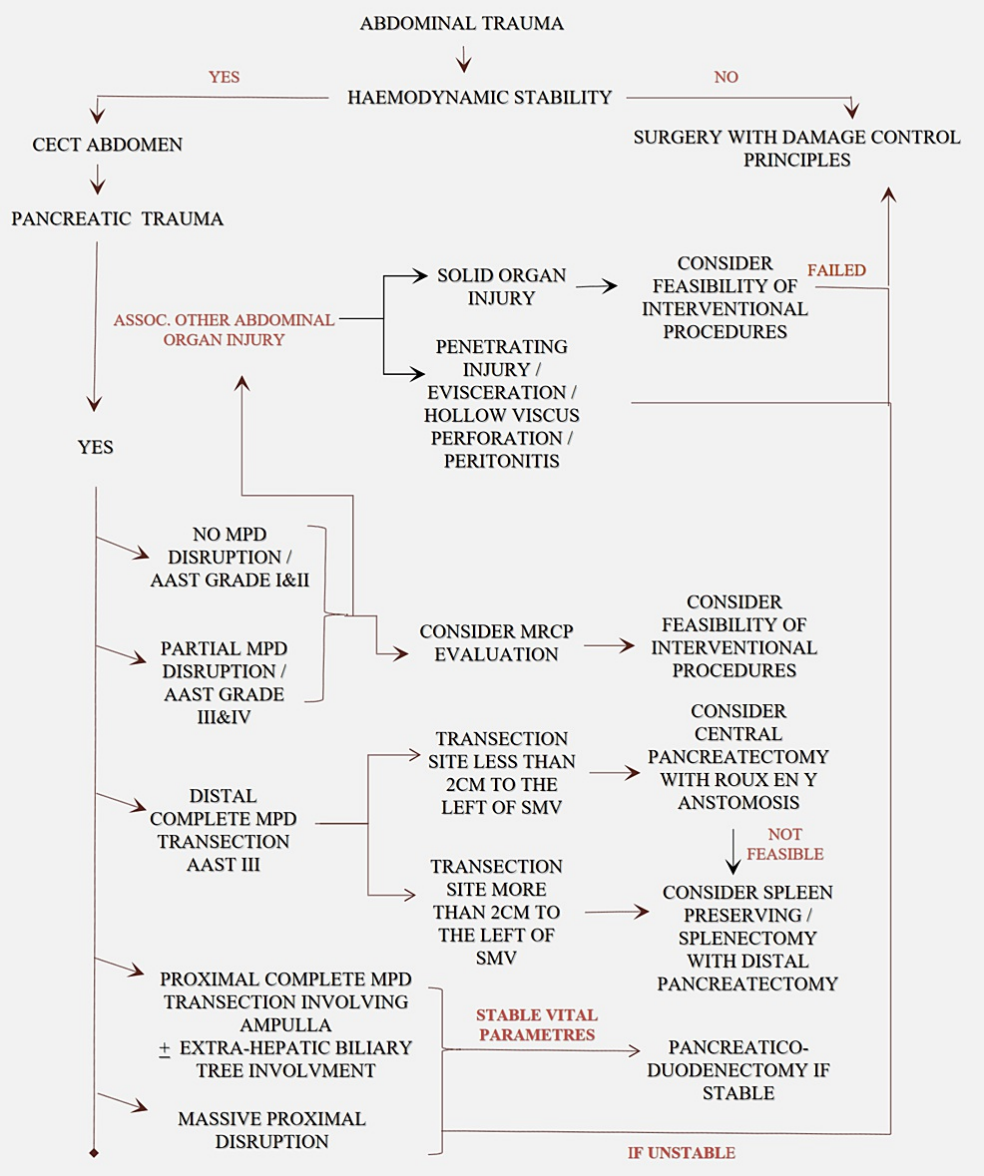
Proposed algorithm for management of pancreatic trauma

**Figure 2:**
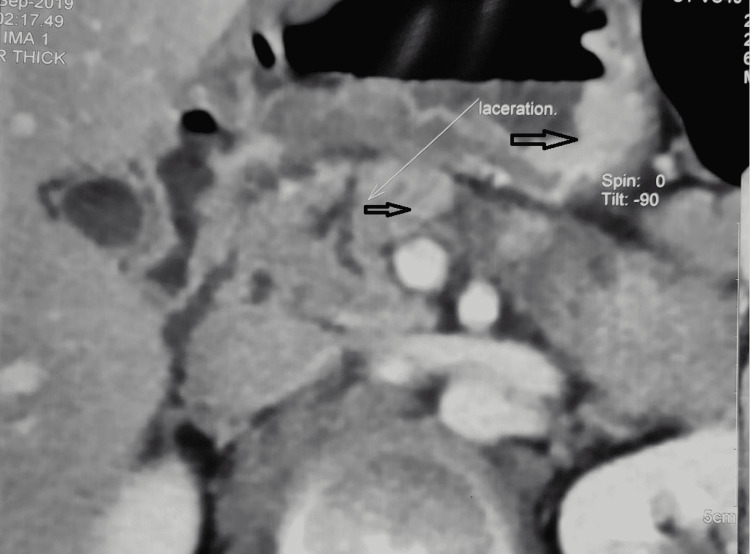
CECT abdomen showing Grade 4 pancreatic injury with lesser sac collection CECT - contrast-enhanced computed tomography; upper black arrow - lesser sac collection; lower black arrow - Grade 4 pancreatic injury

**Figure 3:**
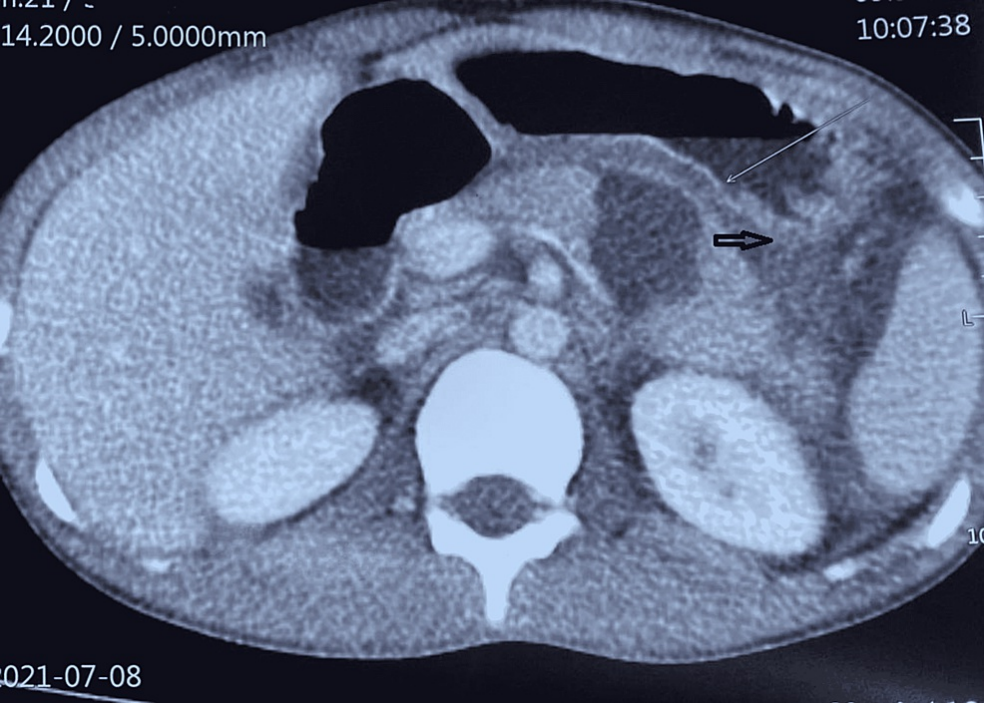
CECT abdomen showing Grade 3 pancreatic body injury with loss of pancreatic tissue CECT - contrast-enhanced computed tomography; black arrow - Grade 3 pancreatic injury

This is an observational study of 28 patients from June 2018 to March 2021 in a gastrointestinal (GI) surgical unit. After stabilization, all patients diagnosed with PT on a contrast-enhanced computed tomography scan (CECT) of the abdomen were included in the study irrespective of their age, sex, mode of injury, or associated organ injury. Then, patients were stratified based on the AAST organ injury score into five grades as represented in Table 1 and managed in accordance with the algorithm in Figure 1. CECT abdomen showing AAST Grade 4 and Grade 3 PT were depicted in Figure 2 and Figure 3, respectively. The primary outcome measure was the success of the proposed mode of treatment in terms of recovery. The secondary outcome measure was morbidity in accordance with the Clavien-Dindo classification and 30 days postoperative mortality.

**Table 1:** AAST grading of pancreatic trauma AAST - American Association for the Surgery of Trauma

GRADING	INJURY	DESCRIPTION
Grade 1	Hematoma / Laceration	Mild contusion without duct injury; Superficial laceration without duct injury
Grade 2	Hematoma / Laceration	Major contusion without duct injury; Major laceration without duct injury or tissue loss
Grade 3	Laceration	Distal transection or parenchymal injury with duct injury
Grade 4	Laceration	Proximal transection or parenchymal injury involving ampulla
Grade 5	Laceration	Massive disruption of the pancreatic head

## Results

The study design was a single-center, retrospective, observational study of 28 consecutive patients who sustained PT. All were males and the mean age of presentation was 33 years.

Mechanism of injury and associated injuries

All sustained a blunt abdominal injury except one. Penetrating injury due to a stab was seen in one patient (1/28). Sixty-one percent (61%; 17/28) sustained injury due to a road traffic accident, 18% (5/28) sustained a handlebar injury, and another 18% (5/28) sustained a blow to the upper abdomen in an assault. The mean duration of presentation after the incident was four days. Six of 28 patients had polytrauma. Another 10 of 28 patients had other, associated intraabdominal organ injuries and the rest (12/28) had isolated PT.

Hemodynamic status on arrival and localization of pancreatic trauma

Eight of 28 patients were hemodynamically unstable on arrival. All of them responded well to initial resuscitative measures. All these patients had either polytrauma or another intraabdominal organ injury, except one who had isolated PT with hemophilia. Eleven of 12 patients with isolated PT were hemodynamically stable at presentation. The localization of PT is given in Table 2. The most common site of injury in our study was in the body of the pancreas (14/28 patients). Five and nine patients had an injury to the head and tail of the pancreas, respectively.

**Table 2:** Baseline population parameters RTA - road traffic accident; AAST - American Association for the Surgery of Trauma

Parameters	Number of patients
Mechanism of injury - RTA	17
Fall from height	5
Blow to the upper abdomen	5
Penetrating (stab)	1
Associated injuries - duodenal injury	3
Splenic injury	3
Gastric injury	3
Liver injury	1
Left adrenal injury	1
Non-abdominal injuries	7
AAST grading - Grade 1	1
Grade 2	2
Grade 3	23
Grade 5	2

Nonoperative management

**Figure 4:**
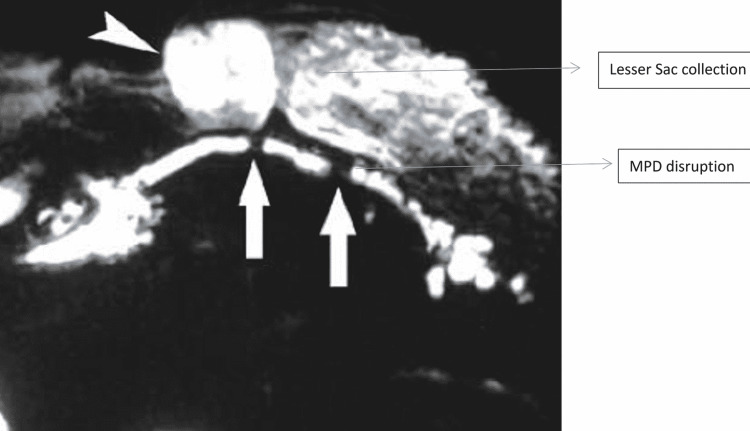
MRCP showing MPD disruption at two places with lesser sac collection MRCP - magnetic resonance cholangiopancreatography; MPD - main pancreatic duct

There were only four patients with isolated PT who were amenable to conservative management. All of them underwent a magnetic resonance cholangiopancreatography (MRCP), in addition to an initial CECT abdomen, to assess the presence and extent of the main pancreatic duct (MPD) disruption (Figure 4). Two patients of Grade 2 and two patients of Grade 3 were managed conservatively. Of the two patients with Grade 2 injury, one had a partial laceration in the head and another at the distal body. Both these patients had features of mild pancreatitis and settled with conservative management. One patient with Grade 3 injury had a complete transection near the tail with a small distal remnant and was relatively stable at initial presentation with only mild pancreatitis. On monthly follow-up for the first six months, he had no pseudocyst formation with progressive atrophy of the distal remnant. Another patient with Grade 3 injury who was managed conservatively had partial MPD disruption in the body of the pancreas with a persistent collection and signs of sepsis beyond two weeks. Ultrasound-guided catheter drainage was done in this patient. He had complete resolution at the six-month follow-up and was operated on for a traumatic lumbar hernia one year later.

Operative management

**Figure 5:**
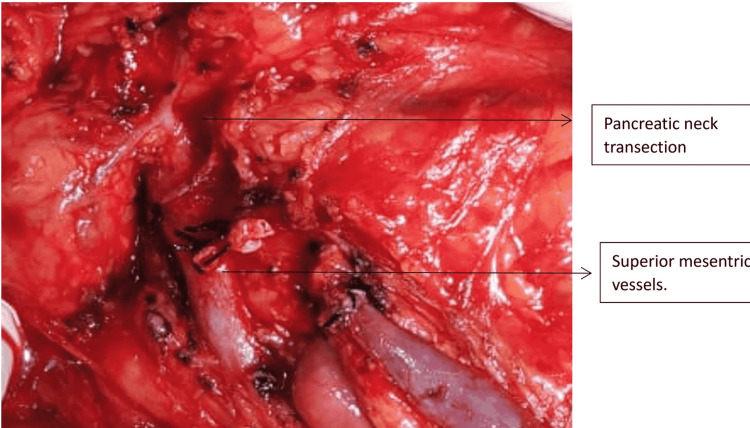
Intraoperative depiction of Grade 5 pancreatic injury with pancreatic neck transection

**Figure 6:**
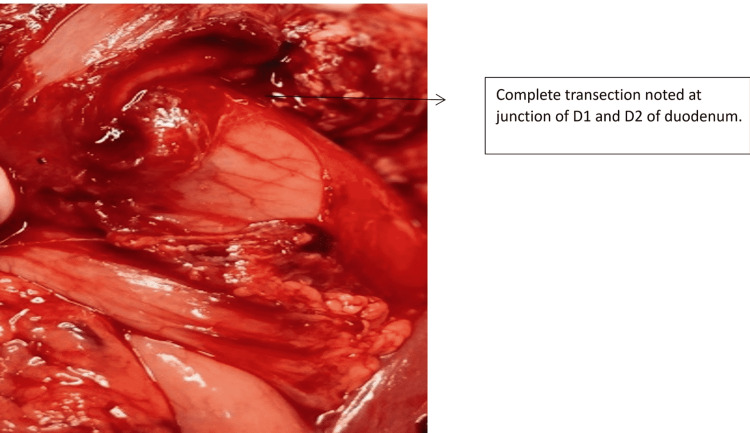
Intraoperative depiction showing a duodenal injury

**Figure 7:**
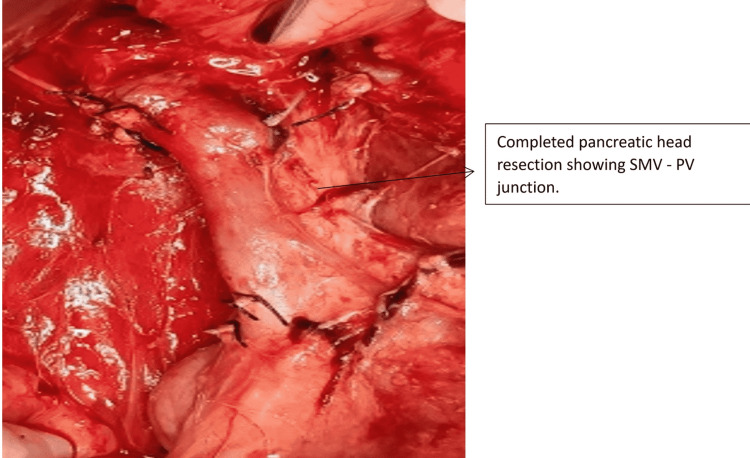
Completed pancreatic head resection SMV - superior mesenteric vein; PV - portal vein

**Figure 8:**
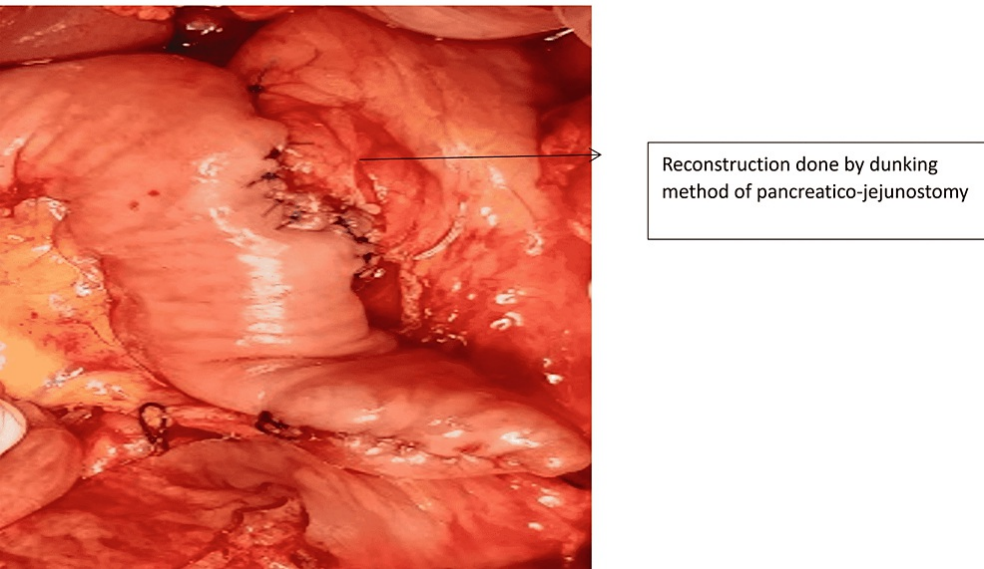
Pancreaticojejunostomy reconstruction done by the dunking method

One patient with Grade 1 injury was operated on for associated hollow viscus injury. Two patients sustained Grade 5 injury due to a steering wheel in a high-velocity crash. Both these cases were hemodynamically unstable at presentation and were referred to us after 48 hours. Both of them responded well to initial resuscitation. In one patient, there was extensive laceration of the pancreatic head with duodenal trauma of the second part of the duodenum as depicted in Figure 5 and Figure 6. As he was stable during surgery without any general peritoneal contamination, we proceeded with a trauma Whipple procedure (Figure 7). In the second case, there was extensive laceration of the head of the pancreas with complete transection at the neck and the first part of the duodenum with Grade 4 splenic laceration. After the initial splenectomy, trauma Whipple was done. In both these cases, we resorted to pancreaticoduodenectomy, as we had the experience and expertise. In both cases, the pancreas was soft and the bile duct was small. The total operating time in both these cases was four and five hours, respectively. The pancreaticojejunostomy was done by a dunking technique in both cases (Figure 8).

**Figure 9:**
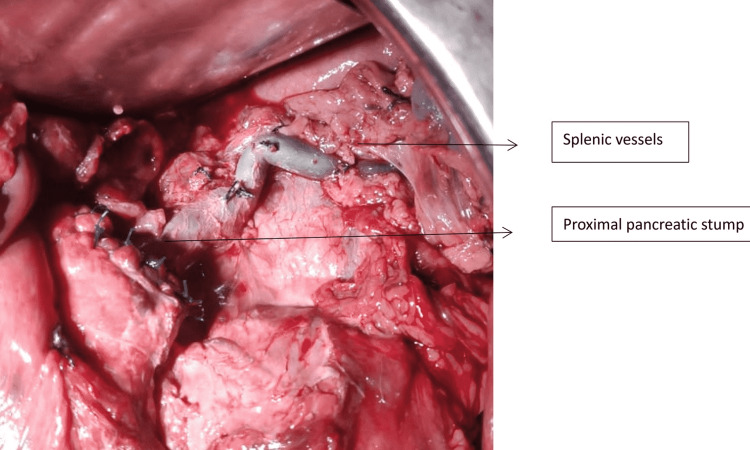
Intraoperative depiction showing completed Kimura’s procedure bearing the splenic vessels

Twenty-one patients with a Grade 3 injury were operated on. Splenic vessel preserving distal pancreatectomy (SPDP) was done in 12 of 21 patients. Distal pancreatectomy with splenectomy and central pancreatectomy with Roux-en-Y PJ was done for seven and two patients, respectively. Various management details in accordance with the AAST grading of injuries are presented in Table 3. A pictorial depiction of completed Kimura’s splenic vessel preserving distal pancreatectomy is given in Figure 9.

**Table 3:** Management details in accordance with AAST grading DP - distal pancreatectomy; DP +SP - distal pancreatectomy with splenectomy; CP + RYPJ - central pancreatectomy with Roux-en-Y pancreaticojejunostomy; AAST - American Association for the Surgery of Trauma

AAST grading	No. of patients	Conservative management	Exploratory laparotomy	
			Surgical DT /Indication for laparotomy	Pancreatic resection
Grade 1	1	----	1 / Gastric antral perforation	
Grade 2	2	2		
Grade 3	23	2	3 (1 - gastric antral perforation ) (2 - referred post-splenectomy )	11 - Splenic vessel preserving DP 5 - DP +SP 2 - CP +RYPJ
Grade 5	2	----		Pancreaticoduodenectomy

Postoperative outcomes

The primary outcome measure of complete recovery was seen in 24/28 patients. The secondary outcome measure of morbidity in terms of Clavien Dindo grades was as follows: Four patients had Grade 1 complications, 12 patients had Grade 2 complications, one patient had Grade 3a, one patient had Grade 3b, four patients had Grade 4a, and one patient had Grade 4b complications. Three died due to polytrauma and pulmonary contusion. Another mortality was seen due to uncontrolled bleeding in a patient with hemophilia. Among the surviving 24 patients, three had pneumonia, seven had surgical site infections (6 SSI and 1 deep SSI), three had DGE, and one patient had a multiorgan failure. An International Study Group of Pancreatic Surgery (ISGPS) Grade A pancreatic leak was seen in 11 patients, a Grade B leak was seen in four, and Grade C was seen in one patient. The patient with Grade C had intense bleeding from the splenic vein on POD 10 for which re-laparotomy and ligation of the splenic vein were done.

## Discussion

The retroperitoneal location of the pancreas makes it less prone to injury either due to blunt or penetrating trauma [9]. But the same makes the diagnosis of PT difficult as well. Due to the close proximity of the pancreas to major visceral and vascular structures, isolated PT is uncommon [10]. A high-velocity injury is required for this deep-seated organ to be injured. Acceleration-deceleration injury and direct compression are mechanisms of blunt pancreatic trauma. Blunt pancreatic trauma often involves the neck and body of the pancreas, as it is immobile and lies on the spine posteriorly. A motor vehicle accident is the most common mode either due to a hit to the epigastrium by a steering wheel or motor bicycle handlebar injuries [11]. The associated organs most commonly injured are the liver (42%) and stomach (40%) in blunt traumas and the colon in penetrating trauma [12]. In our series, road accident was the most common cause followed by handlebar injury and assault. 

The diagnosis of pancreatic trauma requires a high index of suspicion as the clinical features are very subtle in the early stages [13]. Pancreatic trauma shows temporal evolution due to the onset of pancreatitis which leads to a self-amplifying sequence of events. PT should be suspected in all patients with high-velocity injuries and polytrauma [14]. PT should be ruled out in all patients with a history or clinical evidence of the upper abdominal impact of any form. The triad of upper abdominal pain, raised leucocyte count, and elevated serum amylase levels are suggestive but can be absent during the first 24 hours and even after that [15]. Computed tomography (CT) is the imaging modality of choice in suspected pancreatic trauma, and it also identifies associated injuries. It has a sensitivity of 60% to 85% in detecting PT [16]. MRCP can be used for visualizing pancreatic ductal integrity [17].

After initial stabilization, a CECT abdomen should be taken, which is the gold standard. A parenchymal laceration involving more than half the pancreas either in the anteroposterior or the superoinferior axis on the CECT abdomen is more likely to be associated with an MPD disruption. However, CT can either miss or underestimate the depth of laceration during the early phases, as the accumulation of fluid within the gap and separation of fragments occurs in a time-dependent phenomenon [18]. In our series, an early CECT abdomen was successful in identifying PT in 82% (23/28 cases). In three patients who had a splenic injury, the initial CT scan could not pick up a distal Grade 3 PT, possibly because the perisplenic hematoma was obscuring the visualization of the tail of the pancreas. In two cases, a high index of suspicion with serial CECT abdomen was able to pick up PT. In general, the sensitivity of the CECT abdomen increases with increasing grades of PT. In low-grade PT (Grades 1 and 2), serial scanning might be needed. A serial CECT abdomen is also necessary for monitoring the evolution of PT in those patients managed conservatively [19]. In low-grade injuries, an MRCP is an adjunct to CT abdomen in ruling out ductal injury. Similarly, an MRCP plays a complementary role in assessing the extent of MPD disruption in Grades 3 and 4 PT, which are managed conservatively. A CECT abdomen is also essential in the grading of PT.

Despite several grading systems, the AAST grading system is pragmatic and guides treatment decisions [7]. Grades 1 and 2 are considered low-grade PT, and the rest as high-grade PT. Grades 3 and 4 are a conglomeration of partial and complete ductal disruptions in the distal and proximal pancreas, respectively. In both these grades, there is a subgroup of patients with partial ductal disruption who can be managed successfully with endotherapy alone. Grade 5 injuries require surgical management.

Low-grade PT is accompanied by pancreatitis. In the absence of associated other organ injuries requiring surgery, patients with low-grade PT can be managed by the step-up approach for pancreatitis. Adequate fluid resuscitation, analgesia, and serial monitoring for local or systemic complications of pancreatitis usually suffice for these patients. After discharge, these patients must have a regular follow-up at periodical intervals.

A selected sub-group of hemodynamically stable patients with isolated Grades 3 and 4 PT with partial ductal disruption, having no features of peritonitis or prolonged paralytic ileus, and tolerating oral diet can be managed by image-guided drainage of any focal collections and endoscopic retrograde cholangiopancreatography (ERCP) stenting of the ductal disruption. These patients require close, serial clinical and radiological monitoring. Though the majority of these patients respond well to the above measures, any sign of deterioration with persistent tachycardia, failure to thrive, or features of abdominal compartment syndrome must prompt surgical intervention.

**Figure 10:**
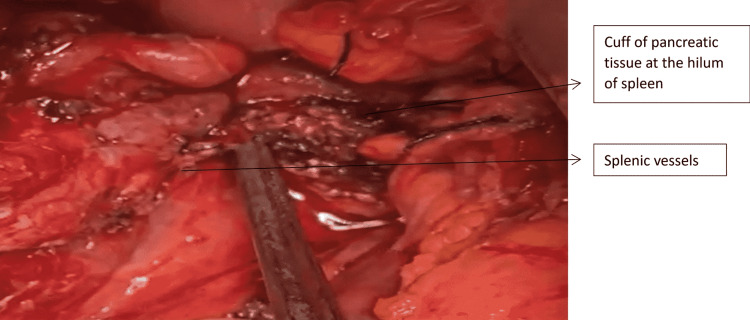
Cuff of pancreatic tissue left intact at the splenic hilum

**Figure 11:**
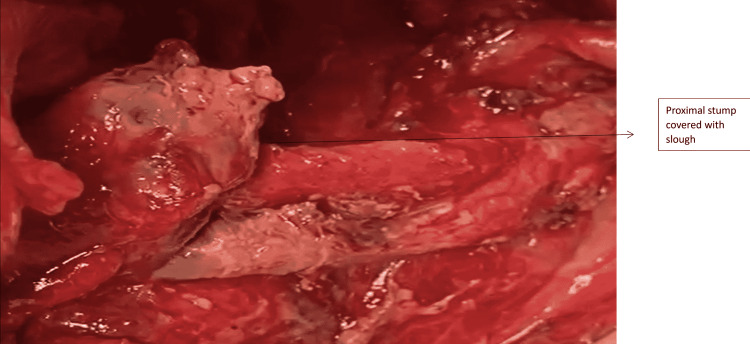
Distal end of the proximal stump obscured due to slough

Complete MPD disruptions almost always require prompt resuscitation and urgent surgical intervention. Distal pancreatectomy is often preferred over drainage because it decreases mortality and the risk of operative complications [20]. In a stable patient with isolated Grade 3 PT, SPDP is a viable option. In such cases, our approach starts with opening the gastrocolic omentum with care taken to preserve two to three short gastric vessels and exposing the site of distal disruption. Then the pancreas is mobilized in a retrograde fashion from the disrupted site distally, exposing the splenic vessels and taking proximal control of them. The use of vessel sealing devices with meticulous dissection helps separate the pancreas from the underlying splenic vasculature. In cases where the splenic vessels are buried within the pancreas, we proceed with the Warshaw technique [21], having already preserved the short gastric vessels. We also found no increase in pancreatic fistulas by leaving a small remnant of the pancreas at the tail near the splenic hilum (Figure 10) by using Kimura’s technique [22]. It is our routine practice to try and close the proximal stump to minimize the chances of Grade B and C pancreatic fistulas. In this series, the proximal stump was closed with sutures in 5/12 cases. In 7/12 cases where the presentation was after four days of trauma, the proximal stump was friable and left without closure (Figure 11). There was no increase in morbidity in these seven cases due to pancreatic fistulas. The postoperative morbidity was significantly lower in SPDP. In two patients with isolated Grade 3 injury and complete distal transection, with the disruption site less than 2 cm to the left of the superior mesenteric vein (SMV)-portal axis; we have done a Roux-en-Y pancreaticojejunostomy. This must be attempted only in a highly select group of stable patients and only in an experienced HPB unit.

Damage control surgery is the dictum in unstable patients with Grades 4 and 5 PT [23]. A trauma Whipple can be done in carefully selected stable patients and where the expertise is available [24]. A trauma Whipple must be carefully considered in a hemodynamically stable patient with massive disruption of the pancreatic head, a severe combined pancreaticoduodenal injury, or destruction of the ampulla of Vater [25]. In patients undergoing a Whipple operation, the bile duct is usually small and the pancreas is soft. In these cases, a dunking type end-to-side pancreaticojejunostomy is our preference and practice. A feeding jejunostomy is our routine practice in patients undergoing trauma Whipple.

The overall mortality and morbidity rates in our series were 15.7% and 64%, respectively, which is comparable to other reported studies. The mortality can be attributed to polytrauma and other associated organ injuries in our series. Morbidity is inevitable in patients with delayed presentation or diagnosis. In our series, postoperative pulmonary complications were very common, especially in those with delayed presentation. Postoperative pancreatic fistula (POPF) and delayed gastric emptying (DGE) are common, especially in those undergoing a Whipple operation, and can be managed according to standard principles.

To summarize our observations, AAST Grades 3 and 4 comprise a composite group with partial and complete MPD disruptions. Partial MPD disruptions amenable to non-surgical modes of treatment irrespective of their location in the pancreas can be given a separate lower order of grading in the AAST system. Distal complete parenchymal transections can be placed next in the order where distal pancreatectomy is effective in an emergency setting. Proximal pancreatic stump suture closure may not be feasible in all cases and there is no increase in the rates of Grades B and C POPF in those where it was not closed. A simple modification of leaving a cuff of pancreatic tissue at the splenic hilum in the SPDP prevents injury to the splenic hilum with no additional morbidity due to pancreatic fistulas. Proximal complete parenchymal transactions or those involving the ampulla or extrahepatic bile duct and massive proximal disruptions warrant the same management based on patient stability.

The limitation of our study is that it is an observational one and hence robust conclusions cannot be implemented.

## Conclusions

The pathogenesis of PT is a dynamic process and shows temporal evolution. These patients require serial and periodical clinical and radiological monitoring, especially in those managed conservatively initially. PT can be low or high grade. Patients with isolated low-grade PT can be managed according to the standard step-up approach for acute pancreatitis. A carefully selected subgroup of patients with partial MPD disruption either in the head or body of the pancreas can be managed by endotherapy. Complete distal parenchymal transections require early surgery tailored to individual patients in the form of either SPDP or DP+S. Damage control surgery is the dictum in unstable patients with Grade 4 and 5 injuries not responding to resuscitative measures. A trauma Whipple can be done in a carefully selected subgroup of stable patients with proximal massive disruptions in an experienced HPB unit.
